# Prediction of corneal astigmatism based on corneal tomography after femtosecond laser arcuate keratotomy using a pix2pix conditional generative adversarial network

**DOI:** 10.3389/fpubh.2022.1012929

**Published:** 2022-09-16

**Authors:** Zhe Zhang, Nan Cheng, Yunfang Liu, Junyang Song, Xinhua Liu, Suhua Zhang, Guanghua Zhang

**Affiliations:** ^1^Shenzhen Eye Hospital, Jinan University, Shenzhen Eye Institute, Shenzhen, China; ^2^Department of Cataract, Shanxi Eye Hospital, Taiyuan, China; ^3^First Hospital of Shanxi Medical University, Taiyuan, China; ^4^College of Biomedical Engineering, Taiyuan University of Technology, Taiyuan, China; ^5^Department of Ophthalmology, First Affiliated Hospital of Huzhou University, Huzhou, China; ^6^Taiyuan Central Hospital of Shanxi Medical University, Taiyuan, China; ^7^Department of Intelligence and Automation, Taiyuan University, Taiyuan, China; ^8^Graphics and Imaging Laboratory, University of Girona, Girona, Spain

**Keywords:** femtosecond laser arcuate keratotomy, corneal tomography, conditional generative adversarial network, surgical planning, deep learning model

## Abstract

**Purpose:**

This study aimed to develop a deep learning model to generate a postoperative corneal axial curvature map of femtosecond laser arcuate keratotomy (FLAK) based on corneal tomography using a pix2pix conditional generative adversarial network (pix2pix cGAN) for surgical planning.

**Methods:**

A total of 451 eyes of 318 nonconsecutive patients were subjected to FLAK for corneal astigmatism correction during cataract surgery. Paired or single anterior penetrating FLAKs were performed at an 8.0-mm optical zone with a depth of 90% using a femtosecond laser (LenSx laser, Alcon Laboratories, Inc.). Corneal tomography images were acquired from Oculus Pentacam HR (Optikgeräte GmbH, Wetzlar, Germany) before and 3 months after the surgery. The raw data required for analysis consisted of the anterior corneal curvature for a range of ± 3.5 mm around the corneal apex in 0.1-mm steps, which the pseudo-color corneal curvature map synthesized was based on. The deep learning model used was a pix2pix conditional generative adversarial network. The prediction accuracy of synthetic postoperative corneal astigmatism in zones of different diameters centered on the corneal apex was assessed using vector analysis. The synthetic postoperative corneal axial curvature maps were compared with the real postoperative corneal axial curvature maps using the structural similarity index measure (SSIM) and peak signal-to-noise ratio (PSNR).

**Results:**

A total of 386 pairs of preoperative and postoperative corneal tomography data were included in the training set, whereas 65 preoperative data were retrospectively included in the test set. The correlation coefficient between synthetic and real postoperative astigmatism (difference vector) in the 3-mm zone was 0.89, and that between surgically induced astigmatism (SIA) was 0.93. The mean absolute errors of SIA for real and synthetic postoperative corneal axial curvature maps in the 1-, 3-, and 5-mm zone were 0.20 ± 0.25, 0.12 ± 0.17, and 0.09 ± 0.13 diopters, respectively. The average SSIM and PSNR of the 3-mm zone were 0.86 ± 0.04 and 18.24 ± 5.78, respectively.

**Conclusion:**

Our results showed that the application of pix2pix cGAN can synthesize plausible postoperative corneal tomography for FLAK, showing the possibility of using GAN to predict corneal tomography, with the potential of applying artificial intelligence to construct surgical planning models.

## Introduction

Among patients undergoing cataract surgery, corneal astigmatism is a sizable component of ametropia, with an estimated 50% of patients with cataract exhibiting more than 1.0 diopter (D) (1). Residual corneal astigmatism ≥ 0.75 D might induce symptomatic blur, reducing uncorrected visual acuity and causing halos and ghosting of images (2). Multifocal or extended depth-of-focus intraocular lens (IOL) technologies with diffractive optics are even less forgiving, and patients receiving such IOLs can be affected by as little as 0.5 D of residual refractive astigmatism (3, 4). In one study comprising over 100,000 individuals undergoing cataract surgery in the UK, 78% had astigmatism of ≥ 0.5 D (5), which is a similar rate (78.79%) in China (6). Postoperative astigmatism is an important cause of emmetropia even after routine surgery (7). Therefore, managing preexisting corneal astigmatism at the time of cataract surgery is critical for achieving excellent visual outcomes and meeting patients' expectations for complete, spectacle-free visual rehabilitation (8).

Over the past decade, manual arcuate keratotomy has been performed to reduce preexisting astigmatism during cataract surgery. However, because of the lack of reproducibility in incision depth, length, and alignment (9), the manual arcuate keratotomy procedure has been associated with unpredictable results and complications (10).

The introduction of femtosecond laser technology to arcuate keratotomy has improved the precision of incision parameters (arc length, diameter, and depth of the incision), with a reported mean difference between the intended and achieved laser incision sizes of 0.1 mm (11). However, most femtosecond laser arcuate keratotomy (FLAK) surgical planning is based on empirical adjustment of the manual arcuate keratotomy nomogram. Postoperative correction results and predictability of residual astigmatism are difficult to achieve in refractive cataract surgery (12, 13).

Based on clinical needs, several studies have been conducted on surgical planning for FLAK (13–17). Most of the studies have applied vector analysis based on surgical outcomes and performed linear regression to produce a nomogram (13–15), including our previous study (16). Operators analyzed the linear relationship between the surgical parameters and results based on the numerical data of the surgical design. However, factors that influence the outcome of FLAK correction are multifactorial and complex. The incision causes an overall change in corneal morphology. However, corneal deformation has non-uniform effects in different corneal zones. Corneal tomography is able to display overall and localized corneal deformation.

We considered the possibility of using corneal tomography, which provides richer information on deformation, as a data source for surgical planning model design. Generative adversarial networks (GANs) (17) are a family of unsupervised machine learning algorithms that have demonstrated their merits by generating synthetic images and solving image-to-image translation problems in the natural image domain (18). By combining surgical parameters to generate a reasonable postoperative corneal tomography, a GAN may be able to achieve this task. To the best of our knowledge, the concept of applying GAN to construct a surgical prediction model for FLAK based on raw data from corneal tomography has not been previously proposed.

This study aimed to evaluate the accuracy of FLAK postoperative corneal tomography generated by applying GAN and to assess the feasibility of applying raw corneal tomography data for the FLAK surgical planning model.

## Materials and methods

### Study population

This study was approved by the institutional review board and adhered to the tenets of the Declaration of Helsinki. After a detailed explanation, informed consent was obtained from each patient prior to enrollment.

This study followed the tenets of the Declaration of Helsinki, in compliance with applicable national and local ethics requirements. The Institutional Review Board of Shanxi Eye Hospital approved this single-center retrospective analysis and waived the need for patient consent. This retrospective study included patients who underwent combined femtosecond laser-assisted cataract surgery and penetrating FLAKs using a LenSx laser (Alcon Laboratories, Inc.) between February 2018 and May 2020 at the Shanxi Eye Hospital.

The inclusion criteria were as follows: patients with cataract and preexisting regular corneal astigmatism who desired to undergo mono- or multifocal IOL implantation and patients completing a 3-month follow-up period. The exclusion criteria were as follows: eyes with irregular astigmatism, keratoconus, and previous corneal refractive treatment.

All patients underwent a full ophthalmological examination. Corneal tomography was performed using an Oculus Pentacam (Optikgeräte GmbH, Wetzlar, Germany). Eye alignment evaluations and measurements with good quality (graded as “ok”) obtained using Pentacam were used in the final analysis. All measurements were performed in a semidark room with undilated pupils.

Paired or single anterior penetrating FLAKs were performed at an 8.0-mm optical zone with a depth of 90% using a LenSx laser (Alcon Laboratories, Inc.). The astigmatic magnitude and axis used for the calculations were determined by the surgeon (SZ) based on preoperative corneal data from Pentacam. The arcuate keratotomy length was determined according to the surgeon's previously published nomogram (17). The arc length ranged from 25 to 65°. After the laser procedure, a main limbal incision was created using femtosecond laser.

### Data preprocessing

We constructed datasets, which collected preoperative and postoperative corneal tomography data of 318 patients; raw data were obtained from Oculus Pentacam HR. It consists of a rotating Scheimpflug camera and the Pentacam software. A total of 318 patients were followed up for 3 months to obtain postoperative corneal tomography data. The data from Pentacam were exported, and the raw data table (CSV format) was imported into the database. The data format was a circle with a radius of 3.5 mm centered on the cornea vertex, and the radius of curvature of each point on the circle was 0.1-mm unit.

**Figure 1:**
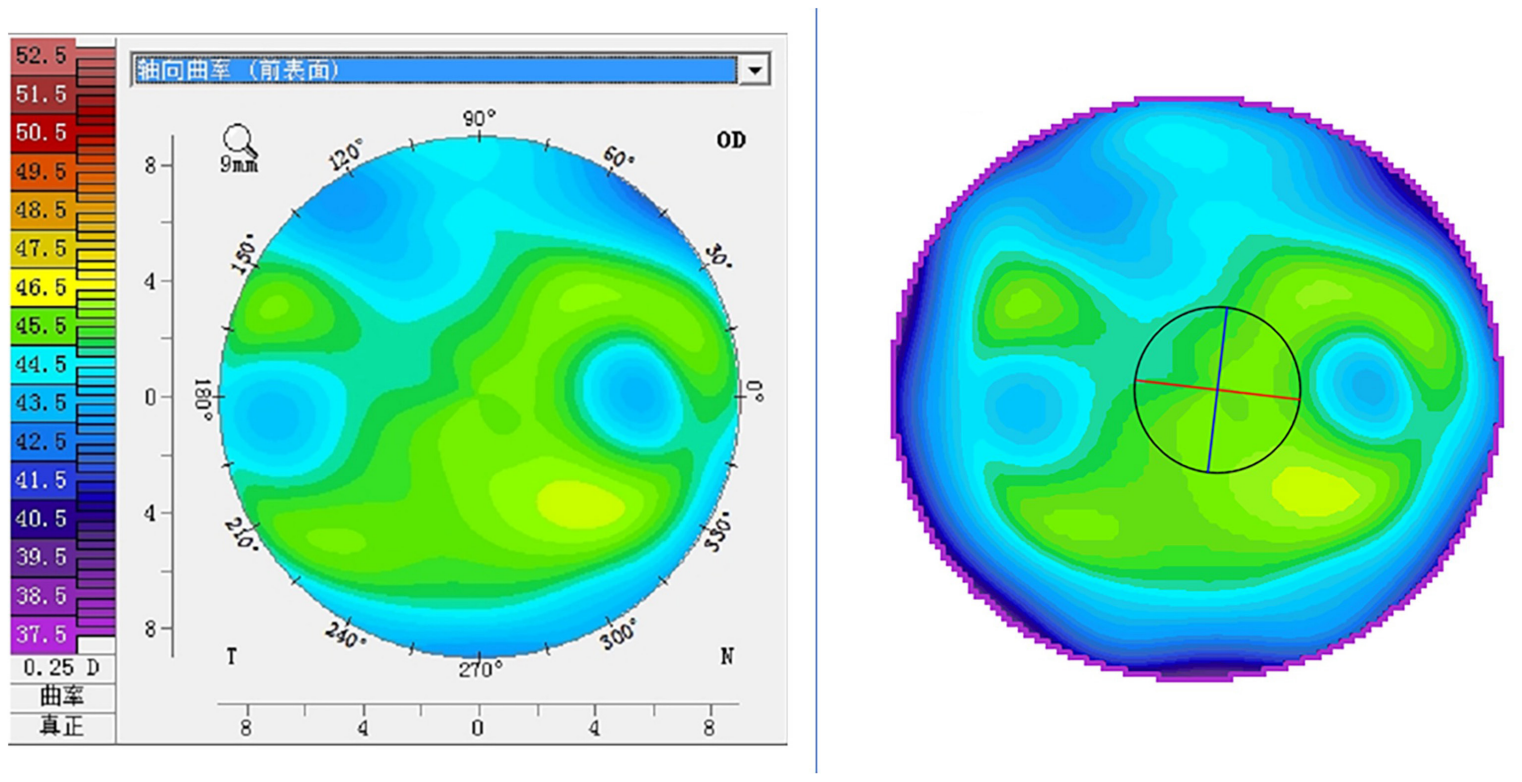
Pseudo-color visualization of corneal tomography: the left image is the axial curvature map derived from the Pentacam; the right image is the pseudo-color map synthesized based on the refractive power values of each point of the cornea (same patient).

**Figure 2:**
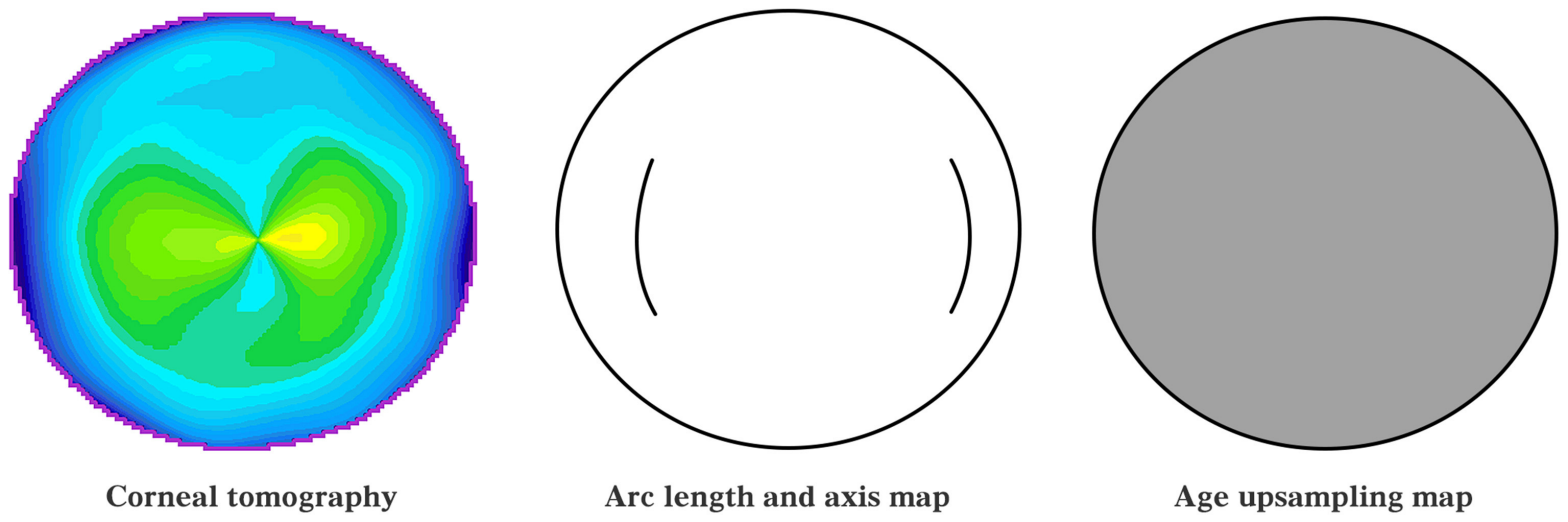
Inputs to the predictive model.

Corneal curvature information was resolved by reading CSV data; subsequently, the curvature data were converted into refractive power data by the conversion formula of radius of curvature and refractive power. Then, corneal tomography could be visualized. The formula can be expressed as follows:


(1) $$\begin{array}{l} Dpt=\frac{\left(1.3375-1\right)\times 1000}{R} \end{array}$$

where Dpt refers to the diopter and R refers to the radius of curvature. Then, the mean value of the refractive power at all points was calculated and ranked to determine the diameter with the highest mean value of refractive power in the 3-mm zone. A Pentacam-like axial curvature map could be displayed, the effect of which is shown in Figure 1. The specific implementation process is as follows:

The inputs to the model included two-dimensional corneal curvature 141 × 141 matrices, one-dimensional AK arc length, AK axis position, and patient age. On the one hand, to ensure the dimensional consistency of the input data, the age was normalized to between 0 and 1 and upsampled into two-dimensional information; the AK arc length and axial position information were used to draw AK arc length and axial position maps (AK incision maps). The corneal curvature of the corneal tomography was normalized between 0 and 1. The normalized corneal curvature information, age information after dimensionalization, and AK position map were concatenated together as the final input of the model, as shown in Figure 2.

### Modeling

A GAN deep learning network was selected as the base model. The training process was divided into two stages: the first stage involved fixing the discriminator, training the generator, and saving the generated data in the buffer pool; if the probability of fake data being similar to real data was low, the discriminator fed the result back to the generator for self-optimization. The second stage involved fixing the generator, training the discriminator, and using the real and fake data in the buffer pool to train the discriminator. The cycle was repeated until the loss functions of the generator and discriminator reached equilibrium as follows:


(2) $$\begin{array}{l} \underset{G}{\min}\underset{D}{\max}V\left(D,G\right)=E_{x\sim p_{data}\left(x\right)}\left[logD\left(x\right)\right]\\ \;+E_{z\sim p_{z}\left(z\right)}\left[log\left(1-D\left(G\left(z\right)\right)\right)\right] \end{array}$$

**Figure 3:**
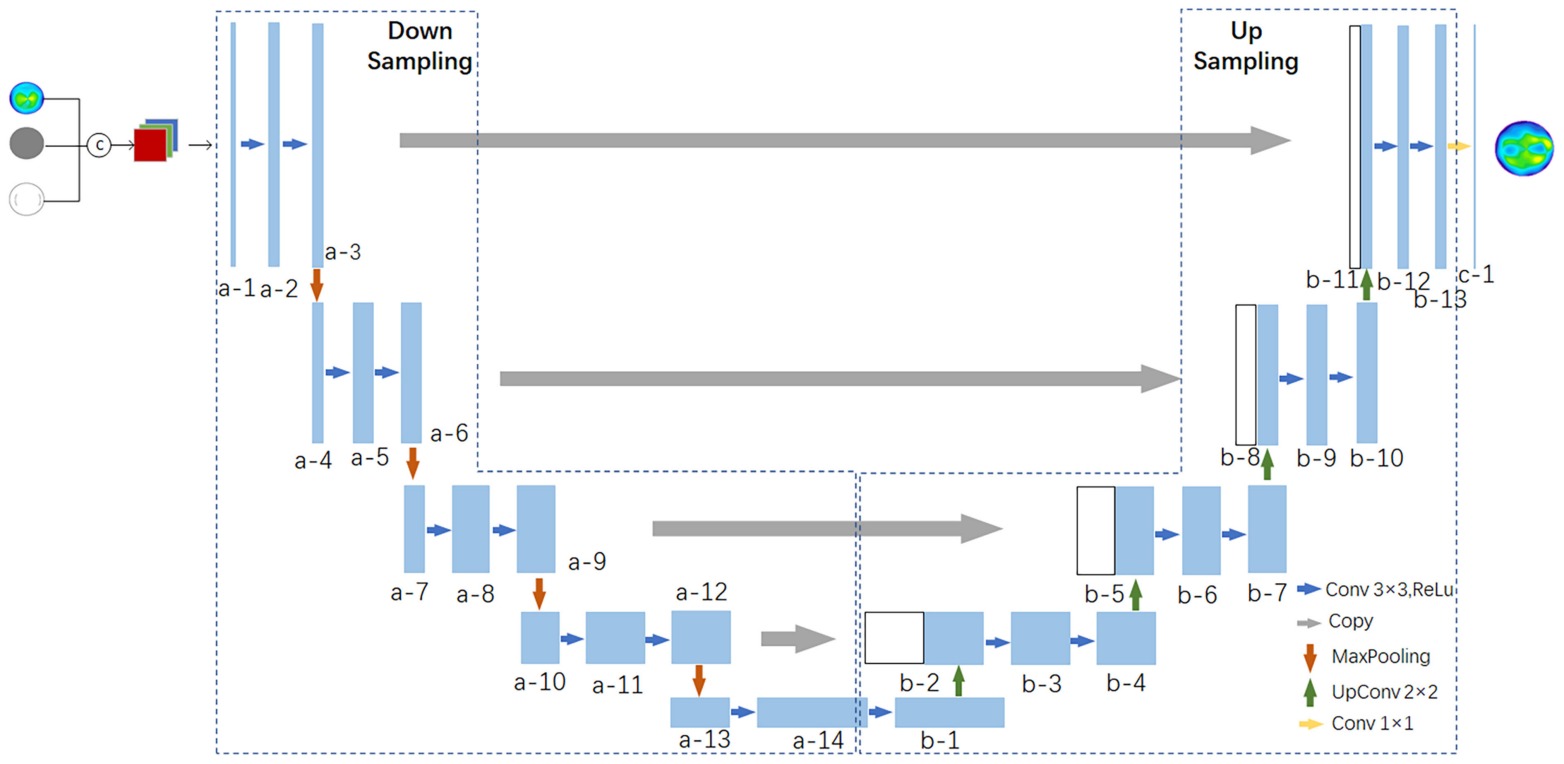
Unet network structure diagram.

The Unet network model was used as the generator of the GAN network to extract the preoperative corneal tomography, age, AK arc length, and other features, which can be used to extract abstract features while preserving more image details with low-level features and can enhance the fineness of the corneal tomography prediction. The detailed structure is shown in Figure 3, in which a multilayer convolutional neural network (CCN) was used to extract the key features of the patient corneal data. In the upsampling stage, the feature maps were upsampled by transposed convolution for image generation. A postoperative corneal tomography prediction map was generated.

**Figure 4:**
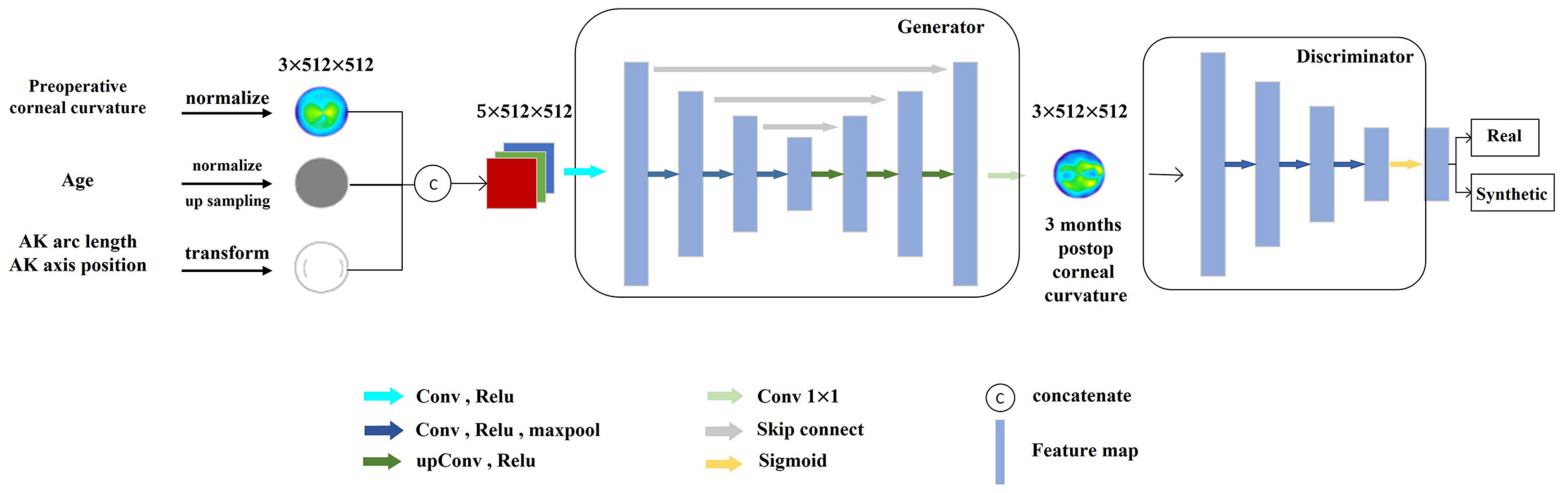
Structure of the postoperative corneal tomography prediction model.

The goal of generating the discriminator module of the adversarial network was to distinguish real corneal tomography from predicted corneal tomography, and the input was either real postoperative or predicted corneal tomography. The loss value of this module can improve the discriminatory ability of the discriminator, and more importantly, the weight value of the generator module can be optimized to improve the quality of the prediction results of the generator module. The prediction model was built using a combination of the two networks (Figure 4).

### Optimizing the training model

The experiments were performed using the Ubuntu 18.04 system, the programming language used was Python 3.6, the deep learning framework was PyTorch, and the hardware environment was i7-8700 CPU and GTX-1080Ti GPU.

In the aspect of data, the accuracy of the model was largely dependent on the quality of the training dataset. This study acquired real medical record images from the clinic and continuously fed new data into the dataset in subsequent clinical applications of the system, thus keeping the model in a constant learning and optimization process.

In the aspect of algorithm, there are many derivative versions of the GAN model. By experimenting with multiple algorithmic approaches and referring to relevant literature to determine the relative optimal solution of the model, we finally settled on using the pix2pix network model as the basis for the experiments. Pix2Pix is an end-to-end method that can perform not only the tasks of a normal GAN model, but also a general method in the field of image generation by introducing conditional parameters to control the mapping of input data to target data. For example, it can render the color of the input image and change the shape of the input image to the shape of the target image.

### Evaluation of model performance

The synthetic postoperative corneal axial curvature maps were compared with the real postoperative corneal axial curvature maps using the structural similarity index measure (SSIM) and peak signal-to-noise ratio (PSNR). The specific formula is as follows:


$$\begin{array}{l} PSNR=10\cdot \log_{10}\left(\frac{MAX_{I}^{2}}{MSE}\right) \end{array}$$

where MAX is the maximum value of the image color data and MSE is the mean square error defined as follows:


$$\begin{array}{l} MSE=\frac{1}{mn}\overset{m-1}{\underset{i=0}{\sum }}\overset{n-1}{\underset{j=0}{\sum }}{\left[I_{real}\left(i,j\right)-I_{fake}\left(i,j\right)\right]}^{2}, \end{array}$$

where *I*_*real*_ and *I*_*fake*_ are the real and generated fake postoperative data, respectively, and m and n are the size of the image. Similar evaluation indices include SSIM, which can be expressed as follows:


$$\begin{array}{l} SSIM\left(x,y\right)=\frac{\left(2\mu_{x}\mu_{y}+c_{1}\right)\left(2\sigma_{xy}+c_{2}\right)}{\left(\mu_{x}^{2}+\mu_{y}^{2}+c_{1}\right)\left(\sigma_{x}^{2}+\sigma_{y}^{2}+c_{2}\right)} \end{array}$$

where x and y are the real and generated fake postoperative data, respectively, μ is the mean value, and σ is the variance. Normally, *c*_1_ and *c*_2_ are constants, $c_{1}={\left(0.01\cdot MAX\right)}^{2}$ and $c_{2}={\left(0.03\cdot MAX\right)}^{2}$. SSIM and PSNR are generally larger and better, where SSIM closer to 1 means that the real data are more similar to the generated data.

The Alpin vector method was used to analyze astigmatism correction outcomes. Target-induced astigmatism (TIA) was defined as the amount of astigmatism the surgeon intended to induce was equal to preoperative corneal astigmatism. The difference vector (DV), defined as the amount of astigmatism that must be corrected postoperatively to finally reach the intended target astigmatism, was equal to the postoperative corneal astigmatism. Surgically induced astigmatism (SIA) represents astigmatic changes achieved by surgery. The correction index was defined as the ratio of SIA to TIA with a value > 1.0, indicating overcorrection, and < 1.0, indicating undercorrection.

The Pearson correlation coefficient was used to compare the synthetic and real DV and SIA. The mean absolute error (MAE) of the SIA was compared between synthetic and real tomography postoperatively.

## Results

A total of 65 pairs of corneal axial curvature matrices were assigned to the test set. In the test set, the mean (standard deviation) astigmatism magnitude was 1.25 (0.52) D in 3-mm zone. The additional baseline clinical characteristics are shown in Tables 1, 2.

**Table 1:** Patient's demographics.

	**Training set**	**Testing set**
**Age (y)**		
Mean ± SD	61 ± 19	59 ± 13
Range	28 to 83	25 to 82
**Single arc length (°)**		
Mean ± SD	44.27 ± 8.26	43.57 ± 9.41
Range	30 to 70	30 to 65
**Paired arc length (°)**		
Mean ± SD	41.23 ± 11.25	42.92 ± 10.00
Range	25 to 65	25 to 65

D, diopter.

**Table 2:** Distribution of preoperative and postoperative astigmatism in different zones of the cornea in the training and test sets.

	**Pre-op**		**Post-op**	
	**Training set**	**Testing set**	**Training set**	**Testing set**
**1-mm zone**				
**K**_**flat**_ **(D)**				
Mean ± SD	43.36 ± 1.71	43.61 ± 1.57	43.44 ± 1.78	43.78 ± 1.55
Range	38.53 to 47.21	38.87 to 47.66	39.31 to 47.65	39.67 to 47.89
**K**_**steep**_ **(D)**				
Mean ± SD	44.52 ± 1.44	44.70 ± 1.58	44.29 ± 1.56	44.46 ± 1.55
Range	40.21 to 48.66	40.77 to 48.73	39.48 to 48.23	39.93 to 48.35
**Astigmatism magnitude (D)**				
Mean ± SD	1.15 ± 0.50	1.10 ± 0.51	0.53 ± 0.72	0.69 ± 0.47
Range	0.37 to 2.64	0.23 to 2.54	0.00 to 2.41	0.02 to 2.34
**3-mm zone**				
**K**_**flat**_ **(D)**				
Mean ± SD	43.35 ± 1.70	43.60 ± 1.58	43.41 ± 1.76	43.90 ± 1.53
Range	38.63 to 47.19	38.70 to 47.59	39.52 to 47.41	39.89 to 48.08
**K**_**steep**_ **(D)**				
Mean ± SD	44.61 ± 1.45	44.85 ± 1.57	44.32 ± 1.57	44.53 ± 1.54
Range	40.23 to 48.72	41.03 to 48.77	39.83 to 48.29	40.18 to 48.18
**Astigmatism magnitude (D)**				
Mean ± SD	1.26 ± 0.53	1.25 ± 0.52	0.71 ± 0.44	0.63 ± 0.38
Range	0.35 to 2.67	0.29 to 2.59	0.32 to 2.09	0.03 to 1.84
**5-mm zone**				
**K**_**flat**_ **(D)**				
Mean ± SD	43.38 ± 1.63	43.63 ± 1.60	43.39 ± 1.63	44.01 ± 1.53
Range	38.72 to 47.16	38.74 to 47.57	39.34 to 47.54	40.05 to 48.08
**K**_**steep**_ **(D)**				
Mean ± SD	44.60 ± 1.44	44.85 ± 1.58	44.31 ± 1.45	44.58 ± 1.55
Range	40.32 to 48.76	41.13 to 48.77	39.89 to 48.60	40.52 to 48.44
**Astigmatism magnitude (D)**				
Mean ± SD	1.26 ± 0.54	1.22 ± 0.52	0.65 ± 0.26	0.57 ± 0.34
Range	0.25 to 2.53	0.16 to 2.41	0.11 to 2.37	0.07 to 1.64

K_flat_, corneal refractive power of the flat axis of corneal astigmatism; Ksteep, corneal refractive power of the steep axis of corneal astigmatism; D, diopter.

### Vector analysis of real and synthetic postoperative corneal axial curvature

**Figure 5:**
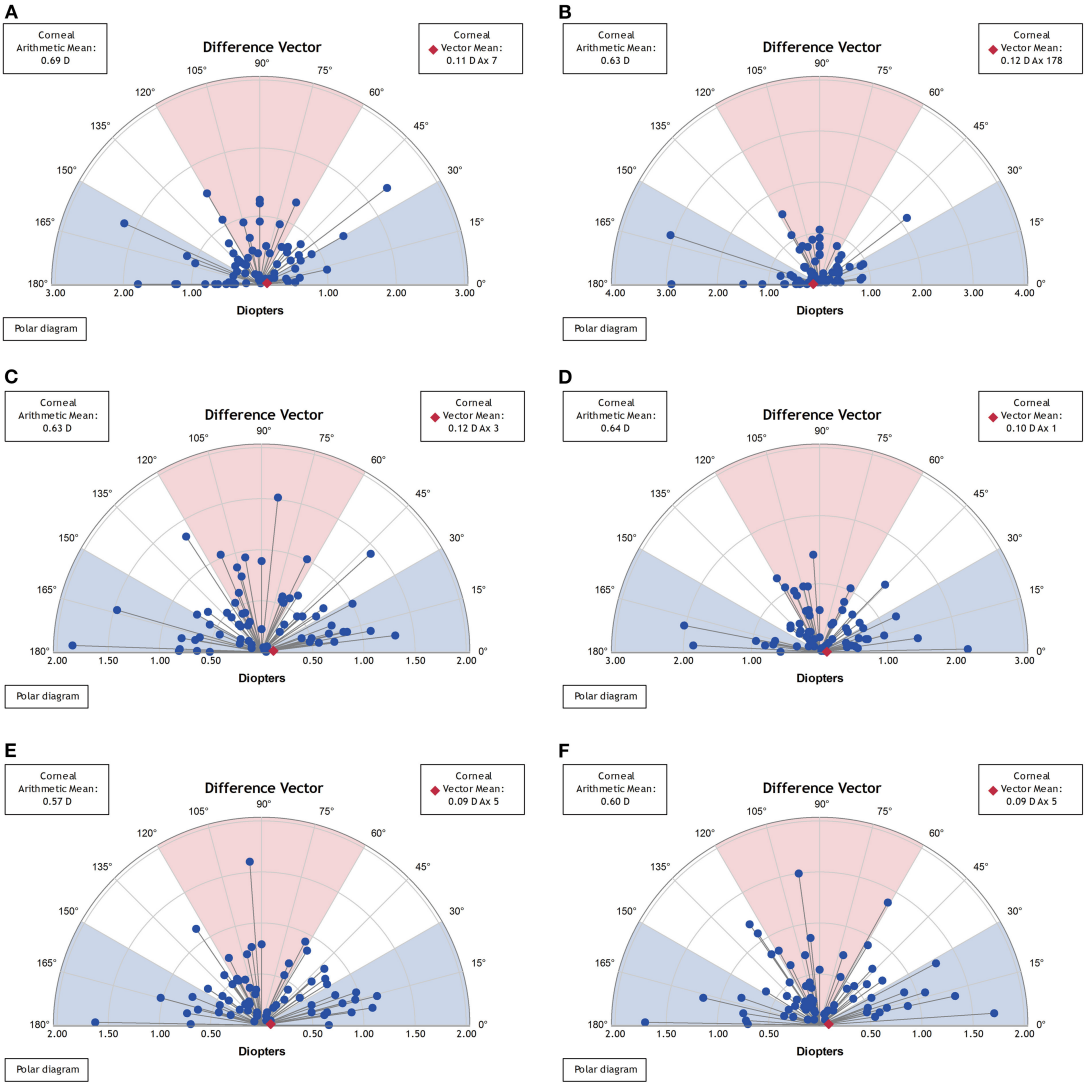
Vector analysis of postoperative real and synthetic DV in the 1-mm, 3-mm, and 5-mm zones. **(A)** The real postoperative DV of the 1-mm zone. **(B)** The synthetic postoperative DV of the 1-mm zone. **(C)** The real postoperative DV of the 3-mm zone. **(D)** The synthetic postoperative DV of the 3-mm zone. **(E)** The real postoperative DV of the 5-mm zone. **(F)** The synthetic postoperative DV of the 5-mm zone.

Vector analysis of postoperative real and synthetic DV in the 1-, 3-, and 5-mm zones: 0.69 ± 0.47 and 0.63 ± 0.57 D for real and synthetic astigmatism in the 1-mm zone (*p* = 0.200), 0.63 ± 0.38 and 0.64 ± 0.47 D in the 3-mm zone (*p* = 0.598), and 0.57 ± 0.34 and 0.60 ± 0.40 D in the 5-mm zone (*p* = 0.218) (Figure 5 and Table 3).

**Table 3:** Vector analysis of real and synthetic postoperative corneal axial curvature in different diameter zone.

**Vector analysis parameters**	**1-mm zone**			**3-mm zone**			**5-mm zone**		
	**Real**	**Synthetic**	***P***	**Real**	**Synthetic**	***P***	**Real**	**Synthetic**	***P***
**DV**									
Arithmetic mean ± SD, D	0.69 ± 0.47	0.63 ± 0.57	0.200	0.63 ± 0.38	0.64 ± 0.47	0.598	0.57 ± 0.34	0.60 ± 0.40	0.218
Range, D	0.02 to 2.34	0.04 to 3.08		0.03 to 1.84	0.03 to 2.27		0.07 to 1.64	0.07 to 1.72	
**SIA**									
Arithmetic mean ± SD, D	1.00 ± 0.56	1.01 ± 0.60	0.683	1.05 ± 0.52	1.09 ± 0.55	0.081	1.02 ± 0.51	1.05 ± 0.53	0.135
Range, D	0.12 to 2.68	0.33 to 3.63		0.12 to 2.39	0.26 to 2.78		0.03 to 2.38	0.05 to 2.36	
**CI**									
Arithmetic mean ± SD, D	1.04 ± 0.69	1.06 ± 0.77	0.821	0.91 ± 0.44	0.96 ± 0.58	0.102	0.89 ± 0.43	0.93 ± 0.62	0.210
Geometric mean	0.86	0.89		0.81	0.85		0.79	0.82	
Range, D	0.13 to 4.00	0.27 to 5.42		0.15 to 2.85	0.28 to 3.61		0.11 to 3.07	0.17 to 5.16	

Paired-Samples T Test.

SIA, surgically induced astigmatism; DV, difference vector; CI, correction index; SD, standard deviation.

**Figure 6:**
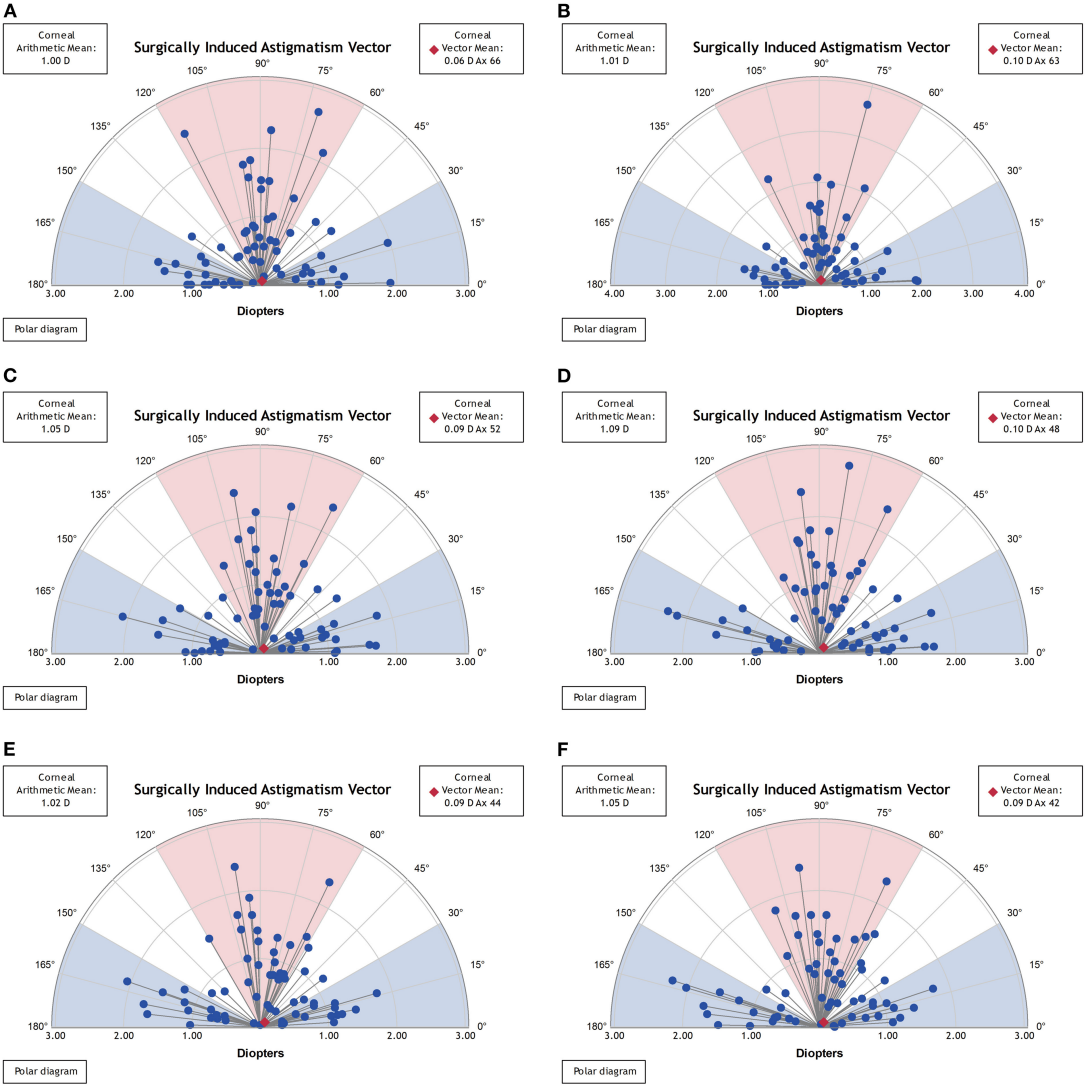
Vector analysis of postoperative real and synthetic SIA in the 1-mm, 3-mm, and 5-mm zones. **(A)** The real postoperative SIA of the 1-mm zone. **(B)** The synthetic postoperative SIA of the 1-mm zone. **(C)** The real postoperative SIA of the 3-mm zone. **(D)** The synthetic postoperative SIA of the 3-mm zone. **(E)** The real postoperative SIA of the 5-mm zone. **(F)** The synthetic postoperative SIA of the 5-mm zone.

Vector analysis of real and synthetic SIA for 1-, 3-, and 5-mm zones: 1.00 ± 0.56 and 1.01 ± 0.60 D for 1-mm zone real SIA and synthetic SIA (*p* = 0.683), 1.05 ± 0.52 and 1.09 ± 0.55 D for the 3-mm zone (*p* = 0.081), and 1.02 ± 0.51 and 1.05 ± 0.53 D for the 5-mm zone (*p* = 0.135) (Figure 6 and Table 3).

**Figure 7:**
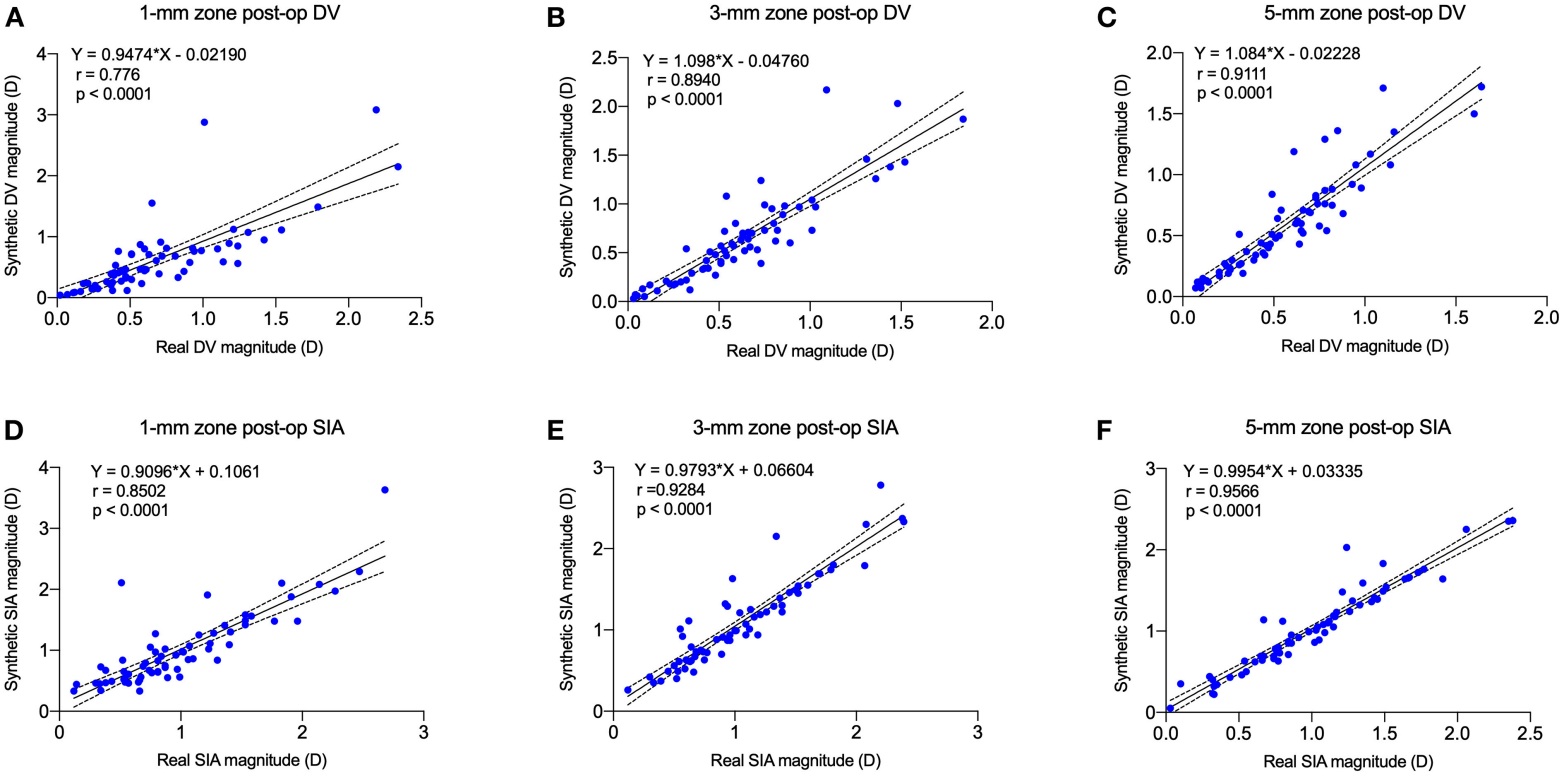
Postoperative real vs. synthetic DV and real vs. synthetic SIA correlation graph for 1-mm, 3-mm, and 5-mm zones. **(A)** The correlation between real and synthetic DV in the 1-mm zone. **(B)** The correlation between real and synthetic DV in the 3-mm zone. **(C)** The correlation between real and synthetic DV in the 5-mm zone. **(D)** The correlation between real and synthetic SIA in the 1-mm zone. **(E)** The correlation between real and synthetic SIA in the 3-mm zone. **(F)** The correlation between real and synthetic SIA in the 5-mm zone.

The correlation between real and synthetic postoperative astigmatism (DV) in the 1-, 3-, and 5-mm zones and the correlation graph between real and synthetic SIA (Figure 7): the correlation coefficient between synthetic and real postoperative astigmatism (DV) in the 3-mm zone was 0.89, and the correlation coefficient between SIA was 0.93.

The MAEs of SIA for real and synthetic postoperative corneal axial curvature maps in the 1-, 3-, and 5-mm zones were 0.20 ± 0.25, 0.12 ± 0.17, and 0.09 ± 0.13 D, respectively.

### Objective image evaluation metrics

**Figure 8:**
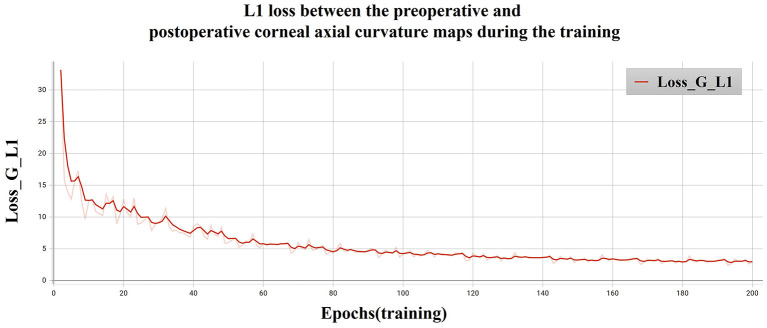
L1 loss between the preoperative and postoperative corneal axial curvature maps during the training.

**Figure 9:**
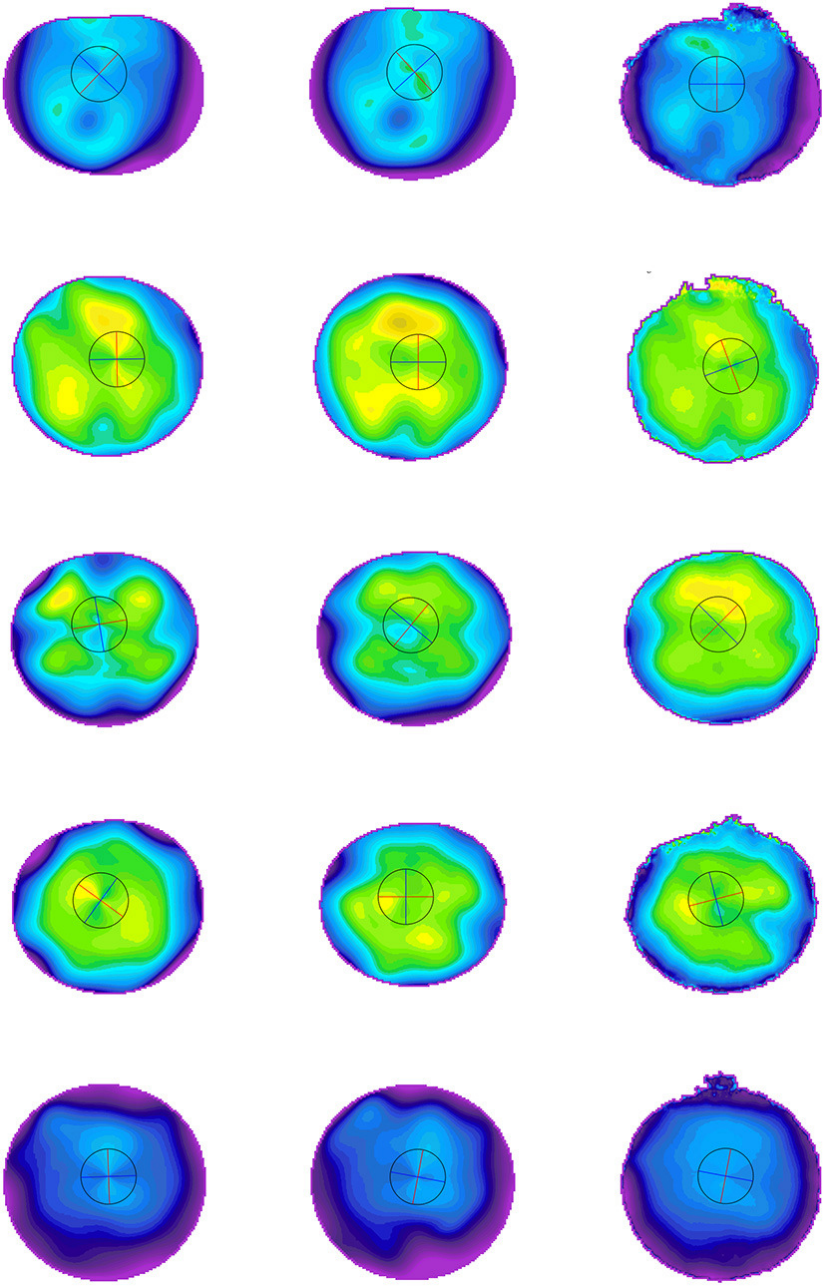
Preoperative and postoperative corneal tomography of five different cases. The first column is the preoperative corneal tomography, the middle is the real postoperative corneal tomography, and the last column is the synthetic corneal tomography.

The L1 loss of the model training is shown in Figure 8. The PSNR and SSIM results for different corneal zones are listed in Table 4. The average SSIM and PSNR of the 3-mm zone were 0.86 ± 0.04 and 18.24 ± 5.78, respectively. The results show that the corneal axial curvature generated by the model after FLAK can simulate the real corneal axial curvature. Preoperative corneal tomography and postoperative real and synthetic corneal tomography images are shown in Figure 9.

**Table 4:** PSNR and SSIM of different corneal zone.

	**PSNR**	**SSIM**
1-mm zone	18.20 ± 6.34	0.72 ± 0.13
3-mm zone	18.24 ± 5.78	0.86 ± 0.04
5-mm zone	17.71 ± 4.70	0.89 ± 0.03

## Discussion

Our present study applied pix2pix to raw data derived from Scheimpflug, which can generate reasonable (plausible quality) corneal tomography after FLAK and shows promising applications for further construction of personalized surgical planning models. To the best of our knowledge, this study is the first essential step in investigating the possibility of using GANs to generate postoperative corneal tomography maps for FLAK planning.

Three main approaches have been used for the surgical planning of FLAK correction of corneal astigmatism.

First, empirical adjustments were performed based on the manual AK nomogram. Ganesh (19) applied the CATALYS femtosecond laser cataract surgery platform to perform penetrating FLAK, and preoperative planning used 8/10 of the recommended arc length of the Donnenfeld manual corneal limbal release incision nomogram (20) as the arc length for FLAK. Wang et al. used 9/11 of its recommended arc length to perform the procedure (16). Baharozian empirically adjusted the Donnenfeld nomogram based on the type of astigmatism to eliminate the effect of posterior corneal surface astigmatism on correction outcomes (13). Lüdeke et al. (3) and Chan et al. (21) used the Wallace and Woodcock manual AK nomogram for surgical planning, with a correction based on postoperative results, and the AK arc length was only correlated with preoperative astigmatism. Löffler et al. (22) used the manual AK nomogram designed by Wang (23), which added the effect of patient age and astigmatism type on correction effects.

All the above surgical planning was based on clinical experience of adjustment or direct application of the manual AK nomogram, with limited predictability of the correction outcomes. A more precise nomogram for surgical planning must be established for the clinical application of FLAK (12, 15).

Second, based on the postoperative correction effect of FLAK, linear regression was applied to correct the surgical planning nomogram by applying vector analysis. In 2016, we performed femtosecond laser penetrating AK based on the Donnenfeld nomogram, using its suggested arc length of 8/11, and performed a vector analysis of postoperative residual astigmatism by applying multiple linear regression to produce a nomogram, which was used to plan the AK arc length by three influencing factors: type of astigmatism, age, and target correction (16). In 2019, Visco used the Nichamin–Woodcock nomogram to perform surgery on 189 eyes, which was also based on vector analysis of postoperative outcomes, to amend the nomogram and add surgical planning for the younger age group (21–45 years) (15). In 2021, also based on linear regression, Wendelstein modified the nomogram, the influencing factors were only arc length and age. However, the type of astigmatism was not analyzed (4). The surgeon analyzed the linear relationship between the surgical parameters and outcomes based on the numerical data for surgical design. However, factors influencing the correction effect of FLAK were multifactorial and complex. The corneal deformation caused by the incision is different from the central to the peripheral cornea, which is not a linear change and therefore leads to poor prediction of the surgical result.

Third, the model for planning FLAK was based on a corneal biomechanical finite element model. Day performed FLAK based on the Stevens' nomogram (24) and found that corneal biomechanical properties had an independent influence on the correction outcome (25, 26). Byun's findings corroborated with Day's analysis (27). Truffer proposed a finite element model based on corneal biomechanics for femtosecond laser intrastromal AK planning, where different surgical parameters can be simulated using this computer model to determine the optimal surgical plan (12). This model demonstrated the possibility of integrating a simulation model to correct corneal astigmatism.

Considering that FLAK incision leads to redistribution of corneal tissue stress and different deformations in different zones of the cornea, an average value of astigmatism cannot be used as the correction target to plan the surgery. Therefore, our study proposes to apply the corneal axial curvature map containing all corneal aberration information as raw data for FLAK surgical planning, which is more accurate. Therefore, our study applied deep learning techniques to analyze the pattern of changes in surgical parameters and corneal tomography for the prediction of postoperative corneal tomography.

Artificial intelligence has been widely used in the medical field. The development of deep learning techniques, particularly CNNs and GANs, can effectively analyze image information. The GAN model has received significant attention since its appearance in 2014 (17), and it can accomplish graph-to-graph, text-to-graph, and text-to-text generations. Therefore, it is widely used for data augmentation and image prediction to extract mathematical relationships from data distribution to match input and output data and automatically synthesize medical images. It is implemented using a system of two neural networks competing in a two-player zero-sum game: discriminator and generator networks. The integrated network performance effectively generates new, plausible image samples. Ophthalmology-related studies have also been progressively conducted and reported in the fields of image synthesis for retinal optical coherence tomography (OCT) (28), anterior segment OCT (29), ocular surface images (30), and corneal tomography (31). Currently, the main algorithms include progressive GANs, conditional GANs (cGANs), image-to-image GANs (pix2pix), and cyclic GANs. Among them, the most widely used GAN architecture is pix2pix, which contains an image-to-image transformation framework (18). The generator part of the pix2pix network uses a U-Net network structure to generate synthetic images directly from the input images by means of layer-hopping connections, which can achieve better generation results. Son et al. (32) applied an algorithm similar to the pix2pix architecture that improves the segmentation accuracy of retinal vessels and optic discs in fundus images. Yu et al. applied pix2pix to synthesize realistic color fundus photographs to expand the dataset (33). Pix2pix has also performed well in segmenting the nerve fiber layer, Bruch's membrane, and choroid-scleral border of retinal OCT images (34).

Liu et al. showed that the pix2pix model can generate post-injection OCT images using pre-injection images for early prognostic prediction (35). cGAN was applied by Lee et al. and trained on pre-injection and post-injection OCT images and fluorescein angiography and indocyanine green angiography images to predict changes in post-injection OCT images (36) to assist clinicians and patients in understanding the effect of interventions on disease prognosis (37). These studies demonstrate the potential of GAN networks for generating high-quality prognostic images.

Abdelmotaal et al. applied a domain-specific CNN based on color-coded Scheimpflug to discriminate keratoconus, subclinical keratoconus, and normal corneal images at levels that may be useful in clinical practice when screening candidates for refractive surgery (38). They used pix2pix to synthesize corneal tomography images, which can overcome issues related to small datasets and class imbalance when training computer-aided diagnostic models (31). These studies demonstrated the possibility of using pix2pix to generate corneal tomography. Different from our study, they input the image information directly, whereas we input the curvature value matrix of each point. These data do not need to be manually labeled, which ensures data standardization and improves the efficiency and accuracy of data acquisition.

The limitations of this study include the fact that the model was developed without applying corneal biomechanical parameters, which are considered one of the factors influencing the outcome of FLAK correction, although age parameters were incorporated. In addition, this model did not incorporate FLAK procedures with different arc incision diameters and depths, and the applicability of the model requires further extension. A follow-up study was conducted based on expanding the amount of data to include corneal biomechanical parameters and finite element models and incorporating data from different surgical parameters and femtosecond laser surgical platforms for model construction to further improve the accuracy of surgical prediction.

In this study, we applied a pix2pix network to construct a postoperative prediction model for the FLAK correction of astigmatism based on corneal tomography features to address the clinical need for a lack of precise surgical planning models. This showed that the model had a significant predictive effect, which verified the validity and feasibility of the algorithm for predicting postoperative corneal astigmatism.

## Data availability statement

The raw data supporting the conclusions of this article will be made available by the authors, without undue reservation.

## Author contributions

ZZ: conception and design, analysis, and interpretation of data, writing the manuscript, critical revision of the manuscript, given final approval. NC and GZ: data preprocessing, optimizing the training model, evaluation of model performance, critical revision of the manuscript, given final approval. YL: data collection, critical revision of the manuscript, given final approval. JS and XL: data collection, analysis and interpretation of data, critical revision of the manuscript, given final approval. SZ: conception and design, performing surgeries, critical revision of the manuscript, given final approval. All authors contributed to the article and approved the submitted version.

## Funding

The work was supported by Shanxi Provincial Department of Science and Technology Research Grant (No. 201601D021142) and partially supported by Shanxi Provincial Health Care Committee Research Fund (No. 2020039), the Fund for Shanxi 1331 Project and the Scientific Innovation Plan of Universities in Shanxi Province (No. 2021L575), the Shanxi Scholarship Council of China (No. 2020-149), and the Salming Project of Medicine in Shenzhen (No. SZSM201812091).

## Conflict of interest

The authors declare that the research was conducted in the absence of any commercial or financial relationships that could be construed as a potential conflict of interest.

## Publisher's note

All claims expressed in this article are solely those of the authors and do not necessarily represent those of their affiliated organizations, or those of the publisher, the editors and the reviewers. Any product that may be evaluated in this article, or claim that may be made by its manufacturer, is not guaranteed or endorsed by the publisher.
